# Anticipating the Future in an Uncertain Present: How Individuals Living With Medically Unexplained Cognitive Complaints Value Biomarker‐Based Prognostics

**DOI:** 10.1111/hex.70284

**Published:** 2025-05-24

**Authors:** Anne‐Fleur van der Meer, Marjan Knippenberg, Denise Visser, Marianne Boenink

**Affiliations:** ^1^ Radboud University Medical Centre, IQ Health, Ethics of Healthcare Group Nijmegen The Netherlands; ^2^ Department of Radiology & Nuclear Medicine, Amsterdam UMC Vrije Universiteit Amsterdam Amsterdam The Netherlands; ^3^ Amsterdam Neuroscience, Brain Imaging Amsterdam The Netherlands

**Keywords:** Alzheimer's disease, biomarkers, individuals' perspectives, medically unexplained cognitive complaints, personalised care, prognostics, responsible innovation, subjective cognitive decline (SCD)

## Abstract

**Introduction:**

In research on Alzheimer's disease (AD) individuals experiencing cognitive problems but not satisfying criteria for mild cognitive impairment (MCI) or AD are referred to as having ‘Subjective Cognitive Decline’ (SCD). A small subset of them will progress to a state justifying an AD diagnosis. AD research trying to identify prognostic biomarkers aims to inform individuals labelled with SCD about the future development of their complaints. This paper explores how persons experiencing these cognitive complaints currently anticipate the future and how they would value emerging biomarker‐based prognostic tests.

**Methods:**

Semi‐structured interviews with 10 individuals experiencing cognitive complaints, some accompanied by their partners. The interviews were coded using ATLAS.ti 22. The exploratory qualitative analysis was conducted using thematic content analysis (TA).

**Findings:**

The interviewees experience pervasive uncertainty concerning their persistent but medically unexplained cognitive symptoms. Lacking a diagnosis and a prognosis, they anticipate their future considering various scenarios, while mainly acting upon the scenario of developing further cognitive decline/AD dementia.

Interviewees often interpret questions about prognostication as asking about predicting the likelihood of a future diagnosis, clearly valuing the latter. Most interviewees are positive, assuming it would help them and their relatives prepare for the future. They have specific ideas about what type of prognostic information would be helpful, usually focusing on how cognitive decline would impact their lives, for example, what activities they will be able to continue to engage in, when they will no longer recognise loved ones or what character they will have as a patient.

**Conclusion:**

People experiencing unexplained cognitive complaints experience pervasive uncertainty about their situation and future. They clearly value the idea of a test predicting with high certainty whether they will be diagnosed with AD in the future. Regarding prognostication, most interviewees expect that such tests could help prepare for the future. However, there is a discrepancy between the outcomes usually measured in prognostic research (like the expected speed of decline) and the specific outcomes meaningful to our interviewees. In view of this gap, it is important to reconsider whether and how the development of prognostic biomarker tests for people suffering from unexplained cognitive complaints can be better tailored to the needs of these individuals.

**Patient or Public Contribution:**

During our interviews, we gathered the perspectives of 10 individuals suffering from medically unexplained cognitive complaints on the value of biomarker‐based prognostics. These interviews were the primary data source. Individuals were also involved in the interpretation of our findings and critical evaluation of our preliminary conclusions.

## 1 Introduction

### 1.1 Aims and Rationale

In the last 10 years, Alzheimer's disease (AD) research has increasingly invested in the identification of biomarkers predicting the evolution of neurodegeneration, leading to AD dementia. Such biomarkers—characteristics that are measured as indicators of normal or pathological biological processes, see Box 1—not only predict who will manifest symptoms but may also prognosticate how symptoms and complaints will develop. Biomarker‐based predictions and prognoses have been produced for various groups, ranging from those already diagnosed with AD dementia, via individuals with ‘Mild Cognitive Impairment’, to healthy, asymptomatic persons.

Box 1. **Biomarkers and their functions**

Definition of biomarkers

The FDA defines a biomarker as ‘a defined characteristic that is measured as an indicator of normal biological processes, pathogenic processes, or responses to an exposure or intervention, including therapeutic interventions. (…) A biomarker is not an assessment of how an individual feels, functions, or survives’ [1].

Testing modalities

The technological modalities involved in measuring biomarkers are wide‐ranging and include imaging and ultrasound technologies scanning various body parts, as well as molecular and biochemical analysis of body fluids (e.g., CSF, blood and urine). More recently, digital technologies like wearables and wet sensors have entered the field, producing digital biomarkers. Biomarker testing may involve measuring a single parameter, but increasingly multiple parameters are combined, resulting in complex composite biomarker profiles [2].

Functions of biomarkers

As set out by Califf [2], biomarkers may serve various purposes, including several that relate to the future of the tested individual. Biomarker testing may establish, among others:

‐ **Risk/susceptibility**: ‘indicating the potential for developing a disease or medical condition in an individual who does not currently have clinically apparent disease or the medical condition’ (p. 217).
‐ **Diagnosis**: ‘detecting or confirming the presence of a disease or condition of interest, or identifying individuals with a subtype of disease’ (p. 214).
‐ **Prognosis**: ‘identifying the likelihood of an event, recurrence or disease progression in patients with a disease’ (p. 217).
‐ **Prediction**: ‘predicting whether an individual or group of individuals is more likely to experience a favorable or unfavorable effect from the exposure to a medical product or environmental agent’ (p. 216).

The difference between prognostic and susceptibility/risk markers is that the former focuses on patients with disease and the latter on healthy individuals without clinically apparent disease. Prognostic biomarkers also differ from predictive ones by identifying the likelihood of different disease states without intervention, whereas the latter establish the likely impact of an intervention in the disease process.

Part of the research has focused on biomarker‐based prognosis for people reporting cognitive complaints, while formal diagnostic testing does not indicate abnormal cognitive functioning, nor provide any alternative explanation for the complaints [3]. In research this group is often labelled as having ‘Subjective Cognitive Decline’ (SCD)—see Box 2.^1^ A small subset of these individuals will progress to mild cognitive impairment (MCI) and AD dementia [3], and this group is therefore seen as an important target group for ‘prognostic’ testing. Such prognostic testing may provide information about (a) the likelihood that one's future condition will justify a diagnosis of MCI and/or AD dementia and (b) how one's complaints and symptoms will evolve (see Box 3 and [3, 4, 5]). This prognostic knowledge is expected to be of great value for the prevention of AD (e.g., ‘SCD individuals are ideal candidates for preventive interventions aimed at delaying and or even preventing AD onset.’ [5, p. S183]), but also for other clinical functions, for example to support personalised care (planning) [3].

Box 2. **Alzheimer's Disease, Mild Cognitive Impairment and Subjective Cognitive Decline.**

The term ‘Alzheimer's Disease’ (AD) for a long time was used as a clinical construct referring to the most frequent subtype of dementia. More recently, ‘AD’ is referred to as the pathology causing dementia, consisting of the formation of amyloid plaques and tau tangles leading to neurodegeneration, while the precise pathological mechanism(s) are as yet unclear [6, 7]. In the past, the presence of pathological plaques and tangles, and hence a definitive AD diagnosis, could be established *post mortem* only. In the last two decades, the emergence of biomarkers has enabled the identification of amyloid and tau pathology, as well as neurodegeneration *In Vivo*, potentially facilitating a more certain diagnosis during life [8].

The observation that individuals may have AD pathology for a long time without experiencing dementia symptoms has spurred extensive research into biomarker‐based prediction and early diagnosis of AD. This has also led to multiple reformulations of disease criteria for (and an increasing fuzziness of terminology surrounding) ‘AD’.

Some argue that ‘AD’ should be approached as a biological, rather than a clinical construct (e.g., [9]). Others propose that AD should be approached as a clinico‐biological entity (e.g., [10]). In any case, the AD process is increasingly conceptualised as a multifaceted, seamless continuum, with individuals moving through subsequent phases [11]:

− The *asymptomatic* phase, in which no symptoms or complaints are experienced;
− The *preclinical* phase in which pathophysiological changes lead to increasing biomarker evidence of ‘AD pathology’, while symptoms are lacking;
− The *symptomatic* phase, subdivided into several stages.
  ◦ First, there are mild and gradually worsening clinical manifestations (a stage often labelled as mild cognitive impairment [MCI]), while biomarker changes continue.
  ◦ The second stage consists of severe clinical manifestations justifying the diagnosis of ‘AD dementia’. Loss of cognitive and overall functioning is increasingly noticeable, ultimately leading to loss of independence and death.

The label ‘Subjective Cognitive Decline’ (sometimes also labelled ‘Subjective Cognitive Impairment’) refers to ‘an individual's perception that their memory or other cognitive abilities are worse than they used to be, without demonstrating any objective neuropsychological deficits’ [12, p. 1090], see also [13]. Although it is not clear whether the experienced decline is associated with biological changes, the reporting of non‐objectifiable symptoms positions SCD between the asymptomatic and the symptomatic phase of the AD continuum. This suggests that SCD may be either a form of ‘preclinical AD’ or the earliest stage of the symptomatic phase, preceding MCI [12].

Box 3. **Prognostic biomarkers for individuals labelled as having ‘SCD’**

Since only a subset of individuals experiencing medically unexplained cognitive complaints (labelled ‘SCD’ in research, see Box 3) progress to MCI and/or AD dementia, biomedical researchers aim to identify biomarkers that predict the future of these individuals. The bodily materials and technological modalities involved include PET scans of the brain and molecular or biochemical analysis of CSF or blood (plasma). The pathological substances tested are similar to those targeted by diagnostic AD biomarkers, including the proteins amyloid, tau and/or various signs of neurodegeneration (often referred to as A/T/N biomarkers).

The biomarkers explored in this research are often referred to as ‘prognostic’. This term may be confusing, however, for several reasons. First, looking at the definitions provided [2, 14], prognostics refers to the future of *patients with a diagnosis*. Since ‘SCD’ is not a recognised disease diagnosis (see Box 2), it would be more apt to call such biomarkers ‘risk/susceptibility markers’. Secondly, prognostic biomarker research in the AD domain pursues a variety of outcome predictions. Whereas a substantial part of the literature focuses on (a) who will ‘convert to’ MCI and/or AD dementia, there is also research predicting (b) how the experienced complaints will evolve (e.g., whether complaints will develop fast or slow, what type of symptoms are likely to develop, how severe these will be and how this will impact one's functioning). Whereas the first aim is about predicting a future diagnosis, the second one is about predicting the future evolution of complaints and symptoms, regardless of whether these will ever justify a diagnosis. The meaning and impact of a prognostic test may substantially vary depending on the type of information provided.

Even though the terminology used in biomedical research does not fully align with the definitions used by the FDA and Califf [2], in this paper we go along with what is common practice in the biomedical (research) domain. Thus, ‘prognostic biomarkers for individuals with SCD’ in this paper refers to biomarkers with either function a or function b above. We discuss them separately where relevant.

If biomarker‐based prognostics are expected to contribute to personalised care, such prognostics need to align with the daily life, perspectives and values of the individuals they are intended to serve. To facilitate such alignment, we need to know what is important for individuals experiencing those cognitive complaints, how they currently view and anticipate their future, and how biomarker‐based prognostics likely would add to and/or interfere with their lives. Therefore, this article reports the findings of a qualitative study exploring how persons experiencing cognitive complaints currently anticipate their future and how they would value such biomarker‐based prognostics. In this article, we explicitly adopt the experiential perspective of the interviewees as our starting point. This means that we do not refer to the interviewees as ‘SCD‐individuals’ or persons ‘with SCD’, as is common in AD research (e.g., [5), but as individuals who suffer from medically unexplained cognitive complaints.

### 1.2 Background

In the medical literature, ‘Subjective Cognitive Decline’ is used to refer to the self‐narrated experience of reduced cognitive function in individuals whose experienced cognitive decline is not objectified during neuropsychological testing [4, 15] and in whom no other standardised neurological or psychiatric diagnosis explains their cognitive complaints [3]. The use of the SCD label has been stimulated by developments in AD research, where AD is increasingly seen as a continuum [7, 11], within which SCD is a phase in the so‐called pre‐dementia stages of AD. In AD drug research, those suffering from SCD are seen as a potential target group for secondary preventive drug trials, because a small subset of this group is at increased risk of progressing towards AD [16]. In many cases, however, the complaints will either disappear or remain stable, while a clear explanation is lacking. The value of the SCD label for clinical practice is, therefore, controversial [15].

Prognostic biomarker tests may help predict how complaints will develop and which individuals in the SCD group will progress towards (‘convert to’) MCI and possibly (AD) dementia. A broad variety of technologies (including neuroimaging and cerebrospinal fluid [CSF] analysis) is currently used to study the potential prognostic meaning of well‐known signs of AD pathology (amyloid, tau and neurodegeneration), both in isolation and combined in multimodal models [12]. The resulting predictions would, first of all, enable more precise inclusion strategies for secondary prevention trials. However, prognostic biomarkers are expected to also provide valuable information for clinical practice. By providing ‘personalised risk scores’, such prognostic testing may not only predict the course of an individual's complaints [17], but could also enable ‘personalised follow‐up, preventive interventions and treatment selection’ [3].

Research into the clinical value of biomarker prognostics for individuals experiencing medically unexplained cognitive complaints is still ongoing [18]. Whether individualised, biomarker‐based predictions will be sufficiently reliable to fulfil the functions mentioned is as yet uncertain. Some sources suggest that individuals testing negative on biomarkers for AD pathology do indeed have a very low risk of conversion to MCI and AD dementia, but the meaning of a *positive* biomarker profile is still unclear [17].

Thus, whether and how prognostic biomarker tests will be able to fulfil the expectations remains to be seen. Even if they will, however, it should not be assumed that intended target groups will appreciate such tests. To assess the value of prognostic tests for people experiencing medically unexplained cognitive complaints, it is important to explore their views early on. To the best of our knowledge, only Mank et al. [19] included individuals ‘with SCD’ in research investigating which prognostic information is relevant to patients and care partners in the full AD disease trajectory (SCD, MCI and AD dementia). However, they do not distinguish between the three subgroups.

Moreover, Mank's study does not take into account that individuals with unexplained cognitive complaints are likely to be engaged with their future. Wondering how their future life will develop, they will be in the process of shaping it, both mentally and practically, and undoubtedly, they are already doing so, even without access to formal prognostic tests.

To understand whether and how biomarker‐based prognostics may be of value to individuals experiencing unexplained cognitive complaints, we need to explore how novel prognostic tools relate to these *current* prognostic practices. We therefore invited them to describe how they *currently* live with their complaints and try to anticipate the future, before asking them how formal prognostic tools like biomarker tests might influence their lives, and how they would evaluate this impact. This approach brings into view the lived reality of people suffering from unexplained cognitive decline, in which prognostic biomarker tools will have to be somehow integrated.

## 2 Methods

### 2.1 Setting

This study was conducted as part of a research project investigating the prognostic value of tau‐PET biomarkers for AD. The biomedical team in particular measured the longitudinal development of tau patterns in people with SCD, MCI and AD dementia and tried to relate these patterns to changes in cognitive functioning and brain atrophy [20, 21, 22, 23, 24, 25]. In addition, a social sciences and humanities (SSH) team conducted qualitative research to explore how different groups across the AD spectrum and their partners value prognostication and the potential introduction of prognostic biomarker tests in clinical practice. In the current study, we aimed to investigate:

1 how people experiencing medically unexplained cognitive complaints value prognostic information (if any) in current practices;
2 how they relate to (imagine, anticipate or deal with) the future and
3 how they look at the potential value of emergent prognostic tools such as tau‐PET imaging.

### 2.2 Data Collection

One of the biomedical researchers (D.V.) contacted individuals considered to have ‘SCD’ in the memory clinic who had already participated in a cohort study and had been subjected to PET scanning and informed them about our interview study. Individuals not able to follow a conversation and give verbal answers, and/or to give informed consent, were excluded. Individuals indicating an interest in the interview study and consenting to transfer of contact details were then further informed (first by written material and then by phone by M.K.) about the aims and set‐up of the interviews. Participants were asked to sign the informed consent form, which was sent via email or post, and return it to the interviewer at the interview. They had over a week to decide before signing. If a partner attended, they also signed the consent form. All signatures were collected before the interview, and permission was recorded on tape under their participant ID.

We ultimately included 10 persons labelled with SCD for the purposes of the cohort study, 5 male and 5 female, with ages ranging from 62 to 76, each of them has a Dutch background. Their complaints had started between 5 and 8 years earlier, and for one person, 13 years before the interview. Interviews took place at the participants' home (*n* = 7), at the memory clinic (*n* = 2) or at our University Medical Center (*n* = 1), and lasted between 30 and 53 min. We left it to participants whether or not to invite a partner to the interview, resulting in 6 interviews with individuals and 4 with couples. To avoid influencing participants' specific ideas about what prognostic knowledge might be possible, we did not mention any specific prognostic outcomes in our interview questions. See Box 4 for participants' prior awareness of SCD, AD and biomarker‐based prognostics.

Box 4. **Participants' awareness of SCD, AD and biomarker‐based prognostics**

**Awareness of SCD**

Participants know from their neurologist that there is no reason to believe that their cognitive complaints indicate Alzheimer's. They might have been told that their complaints can be classified under what is medically referred to as SCD, which stands for ‘subjective cognitive decline’.

**Awareness of AD**

Only general awareness. With the exception of one participant, each of them has known individuals with Alzheimer's dementia within their family, circle of friends or work environment.

**Awareness of therapeutic options**

No specific knowledge of therapeutic options for cognitive complaints or AD.

**Awareness of biomarker‐based prognostics**

The precise prognostic value of the (results of the) biomarker tests participants underwent has not been discussed with them, as the clinical relevance of these tests is not yet known.

Participants do not have specific knowledge about the relationship between the results of (their) biomarker tests and possible future progression of cognitive complaints to MCI or Alzheimer's dementia.

Participants have been told (also in our interviews) in general terms that with help of biomarker tests it might eventually become possible to prognosticate how cognitive function might develop.

### 2.3 Coding and Analysis

All interviews were audiotaped and transcribed and subsequently analysed through Thematic Analysis [26]. The analytical process followed six steps: (1) familiarisation with the data, (2) generating initial codes, (3) developing initial themes, (4) reviewing themes, (5) defining themes and (6) producing the report. The two researchers read all anonymised transcripts and listened to audiotapes (Step 1). The first transcript was independently coded using ATLAS.ti 22 by two researchers (A.M. and M.K.—Step 2). Differences in coding were discussed, and the initial coding scheme was defined. The coding of the subsequent transcripts was distributed among the two researchers. They checked each other's coding in detail. Differences in coding again were discussed, and the coding scheme was adjusted accordingly after each transcript. They used inductive coding (to identify all possible relevant themes) and deductive coding (paying attention to topics immediately relevant to our research questions). After coding the first half of all transcripts, the code set was discussed with the entire SSH research team (A.M., M.K. and M.B.). Finally, A.M. and M.K. reviewed all transcripts for consistency in coding (still Step 2). Regularly, we held meetings with the entire research team, in which Steps 3–5 were carried out: starting from the code set and the research questions, the whole team jointly identified the most striking patterns in the data. These were subsequently used as an analytical lens by A.M. to re‐examine all data (confirming and nuancing early interpretations). This process resulted in the themes presented in this paper (an overview is presented in Table 1).

We shared a summary of our findings and an accessible overview of preliminary conclusions with all interviewees, asking for their feedback and reflections. Five of them sent a response, which we have integrated into the reflection on our findings in the ‘Discussion’ section.

**Table 1:** Themes and sub‐themes derived from interviews with people living with medically unexplained cognitive complaints.

Themes	Sub‐themes
1. Experiences of uncertainty	1a. Uncertainty about the nature of symptoms
	1b. Uncertainty about how to deal with symptoms
2. Trying to anticipate an uncertain future	2a. Awareness of an uncertain future
	2b. Scenario‐thinking
	2c. Scenario‐acting
	2d. Dominance of AD dementia scenario
3. Positive attitudes towards biomarker‐based prognostics	3a. Importance of predictive diagnosis over prognosis
	3b. Reasons for a positive attitude
	3c. Preconditions for a positive attitude
	3d. What people want to know

## 3 Results

### 3.1 Experiences of Uncertainty

Our first finding is that uncertainty is a significant concern for individuals experiencing medically unexplained cognitive complaints (Theme 1). This uncertainty primarily pertains to the nature of their symptoms (Sub‐theme 1a): ‘I just didn't know [what caused it] and I just doubted’ (108). For many, the initial relief when diagnostic tests at the memory clinic show no signs of AD soon gives way to a sense of empty‐handedness and renewed uncertainty: ‘I had no definite answer, what is it, I wanted to know’ (102). Although they are not diseased in the medical sense of the word, their complaints are undeniably present. Most interviewees indicated that the symptoms even worsen over the years. ‘And yes, if you are then told, it is not Alzheimer's, then that is a relief, but the complaints are of course still there’ (107). The uncertainty is also about how to deal with these symptoms, then (Sub‐theme 1b). Contributing to this is that the neurologist offers no information about handling the symptoms after messaging that there is no AD (this was indicated by all our interviewees).

And then I was at a loss as to what to do, because in the end I didn't know what it was either. […] There was nothing wrong, just a few memory complaints. Come on I thought, I had enough peers and my partner at the time. So, it was obvious that something really was not right. […] I didn't really get a good response to that. (110)

Interviewee: [The doctor said:] you don't have to worry, no.

Interviewer: And did they say anything else about how things might go further?

Interviewee: That wasn't necessary. […] ‘Just carry‐on living, Sir’ (109)

### 3.2 Trying to Anticipate an Uncertain Future

A second key theme we identified is dealing with an uncertain future. Each of the interviewees articulates that their future is uncertain, with a small majority (six of them) explicitly indicating that they try to take that uncertain future for what it is (2a): ‘I knew [the diagnostic tests] didn't say everything, so I took it with a grain of salt. We'll just have to wait and see’ (102). They report ‘living by the day’ and having an accepting attitude. They justify this in terms of lacking knowledge (you simply cannot know what the future holds) and the limited influence they have on what will happen.

At the same time, and somewhat self‐contradictory, all interviewees are busy envisioning possible scenarios about their future. The next theme we identified was a constant attempt by our interviewees to envision the future (Sub‐theme 2b: ‘scenario‐thinking’). With the lack of a clear diagnosis and associated probable course of disease, people keep multiple possibilities open: ‘you realize: it could still go off track, right? You never know when it might turn into something worse’ (103). The possibility that their complaints are due to or related to AD dementia plays a major role in these scenarios, particularly if AD dementia runs in the family and especially if individuals have witnessed the impact of AD dementia on patients and their caregivers (Sub‐theme 2d). This is the case even after their diagnostic test showed that there is no reason to think that AD explains the symptoms experienced:

Well, there is no deterioration in it [the complaints]. All tests remain the same, so I assume that it is, in any case, not demonstrable [AD dementia]. Anyway, I also know people who also had it [subjective complaints] and eventually turned out to have Alzheimer's dementia after ten years, so of course it doesn't say everything (102).

‘I do expect the complaints to become more serious and I also consider it possible that I will eventually develop to Alzheimer's disease.’ (107)

All interviewees also try to influence in their *actions* which future will come about (again contrary to the resigned attitude some of them say to have): ‘So right now I'm trying to do things that might prevent it from developing’ (106). We call this scenario‐acting (Sub‐theme 2c). For example, they report eating as healthily as possible (‘Also with your diet, you keep that in mind, not just chewing everything’ (106)), or exercising to prevent worsening of their complaints:

Interviewee: I wait patiently. Keep everything as good as possible, so don't do special things. Just trying to keep up the training, I like puzzles, word games and I read a lot. This is how I try to maintain everything and I exercise and I eat healthy. That won't be the problem.

Interviewer: Could it be that if things are getting worse, that it is more important to know how it will continue?

Interviewee: Well, medically you mean? Yes, but there is not much to say about that. […] There's no point in worrying about it. If it's going to happen, so be it. You can't prevent it. You can, however, ensure that you continue to train your memory and thinking skills. We walk a lot.

Partner: We walk a lot, cycle a lot. So, we do a lot of exercise, et cetera. (103)

The quotes again illustrate the dominance of the AD dementia scenario shaping people's actions: interviewees try to minimise the chance and/or severity of this scenario.

Interestingly, the majority of our interviewees talk about their participation in the cohort study (in which the development of their complaints is tested annually) as a way to monitor their cognitive functioning. Although it rarely is their primary motive for participation, many (seven) mention the ‘indirect control’ of their symptoms as a comforting advantage of participation. Most participants expect that researchers will warn them if the tests show substantial cognitive decline.

Individuals also use their participation for *self*‐monitoring, allowing them to evaluate whether performing the tests was more difficult than in previous years. This is another example of how participants actively engage in their (future) health and well‐being and use their participation for their own anticipatory goals.

### 3.3 Positive Attitudes Towards Biomarker‐Based Prognostics

An important theme is participants' positive attitude towards the idea of being able to know—with help of biomarker tests—how their cognitive problems will develop in the future. Only a few are more sceptical, for example, because they think that their future will be obscure anyway, with or without prognostication (e.g., because formal prognostication provides an *estimate* of *probable* risk only). Others indicate that knowing how one's cognitive complaints will develop obstructs living with an ‘open future’ (103, 105 partner), or fear that the information will be burdensome while living with current complaints is difficult already: ‘why should I already know something when I don't have it yet? It would already put such a limit on your life. Who says it will be like that?’ (103).

To accurately interpret these positive attitudes, it should be noted that all interviewees seem to immediately conceive of the question ‘would you like to know (with biomarker tests) how your symptoms will develop?’ as: ‘do you want to know if you will get Alzheimer's in the future/suffer severe cognitive decline’ (again, see the dominance of that scenario). Prognostication, that is, was understood to fulfil a *predictive diagnostic function* (telling people whether one will experience complaints and symptoms justifying a future diagnosis, Sub‐theme 3a), rather than a prognostic function (understood as forecasting how complaints and symptoms will develop—we elaborate on this distinction in Box 3). Furthermore, individuals assumed that a future biomarker test would unequivocally show *whether* (and *when*) they will develop cognitive decline.

An important reason, according to the respondents, for wanting to know the future is that it aligns with their life attitude and self‐image (Sub‐theme 3b): ‘I am someone who really wants to know things’ (106) or ‘I do [want to know how my complaints will develop]. I have always […] been like, I want to know everything. Because I'm captain of my own ship. […] I don't bury my head in the sand for anything’ (108).

The positive attitudes are motivated by specific and explicitly formulated preconditions (Sub‐theme 3c): (1) the possibility to practically and mentally prepare for AD dementia/cognitive decline, (2) the possibility to prepare a timely euthanasia request in case of anticipating AD dementia^2^ and (3) life planning before the onset of AD dementia. Examples of these preconditions are presented in Table 2.

**Table 2:** Preconditions for a positive attitude.

Preconditions for a positive attitude towards prognostic testing (Sub‐theme 3c)	Example
Possibility of preparation (in general) in case AD dementia is expected	‘That I can say, “well then I'm either just going to spend all my money and then I'll see later” or I'm just going to save everything so I can get help. You know something like that. That you can start thinking about that.’ (102)
Possibility to request euthanasia in a timely manner in case AD dementia is expected	*After talking about the euthanasia of a family member:* Interviewee: ‘And where […] then possibly is the limit? When truly hopeless suffering follows.’ Interviewer: ‘So your sister has arranged that and those are things that you would then also want to arrange yourself.’ Interviewee: ‘Yes. Or have the opportunity to do so.’ (108)
Possibility to plan life before the onset of AD dementia/further cognitive decline.	‘I would still then like to take a trip where I would then like to go. […] A couple of times is enough, so to speak, to see a bit of the world. Just experience that. […] I always have the idea of a helicopter flight. Very silly. But just to say gosh, Rome or something. Seems fantastic, that city trip. But also, say, visiting a really nice warm country or something.’ (108)

We also identified more implicit conditions in their reasoning. For example, half of the interviewees indicate that they like the idea, but they also indicate that much would depend on what information the tests will offer and how ‘certain’ this information will be:

It very much depends on what that says then. If they find something and say: if you have this I'm sure within 4 years you'll be really demented, well then I do want to know, because then I have to know something. But if they say: we found something and now the chance is no longer 1% that you will become demented, but 50. Well, that could take another ten years, yes then I don't know. (102)

Two of them (102 and 103) find prognostics especially valuable if the process of cognitive decline could be *changed* or only if a cure would be available. Another participant says that relevance of prognostics depends on timing: it would be relevant to her only when her complaints would have deteriorated seriously (103).

Interviewees have specific ideas about what kind of information should be predicted (Sub‐theme 3d, summarised in Table 3). The central pattern here is that respondents want to know what cognitive deterioration *will mean* in their lives and relationships. Note that the scenario of Alzheimer's or severe cognitive decline plays a dominant role. For some, prior family or professional experience with the impact of AD dementia motivates what information they would like to have:

Well, if you could predict how it would go, what direction it will develop … so that they say you will have the form in which, for example, you will become very anxious and paranoid or you have a form in which you don't have that at all […] I would like to know, because if I got the form that I would become very anxious and paranoid, well that's awful. Then I just want to die. I don't want to burden myself and the people around me with that, while, if I just get cheerfully demented, well then you all just have to deal with it. (102)

This respondent not only wants to know the nature of her complaints (‘direction’ and ‘form’), but also how she will *feel* and *behave* (‘anxious’ and ‘paranoid’) and *the extent to which* she will feel and behave like that (‘very’ and ‘cheerfully’).

**Table 3:** Types of information about the future considered relevant.

What people want to know (Sub‐theme 3d)	Example
Whether or not their cognitive complaints will develop into AD dementia	Interviewer: ‘So that you could get [AD], that's really most important to you?’ Interviewee: ‘I would want to know that, or well, specifically I want to know if you get Alzheimer's or dementia…. I want to know where those symptoms come from.’ Interviewer: ‘So, then you finally know the cause of those symptoms, that's what [prognostication] would mean for you then.’ Interviewee: ‘Yes.’ Interviewer: ‘Because, now you don't know.’ Interviewee: ‘No, still don't.’ (111)
What will (not) change in their functioning? Individuals mention very concrete things like cooking, driving a car, remembering names of (grand)children and so forth	‘Can you keep functioning a little bit, can you keep cooking food?’ (106, partner) ‘I think ‘the mental’ is the most important thing [to know] for me. Look, if you can't ride a bike anymore, you can always walk. But if you don't *know* anymore, if you can't read a book anymore or you don't know how a coffee machine works or you name it, so you basically just can't go on living.’ (107)
What their Alzheimer's will be like and what ‘character’ they will have as a patient	‘It depends, because you know them. The aggressive dementia patients, loving dementia patients and easy people, you name it. So that just depends on where exactly you end up. But yes, then I hope I don't belong to those very aggressive ones. […] I would find that annoying. If they can predict all that… […] then you have to arrange something sooner, so to speak.’ (110)
How soon their functioning will change, *when* they will lose their memory or *how long* they will still be able to live their life in their current condition	‘Because well then I just want that last six months…. I just want to do other things with my kids or something like that’. (106) ‘I want to know so that […] I can inform my family’: ‘they've told me within now and three years I have early Alzheimer's. Or within now or five years I'll lose my memory. So, then I prepare them, that they are also going to get used to it. It's hard, to accept for a family, it is very hard, but then they know: within now and so much time maybe I don't recognize you anymore, you can stand in front of me and I swear at you all the way like “who are you?”’ (105)

## 4 Discussion

### 4.1 How People With Unexplained Cognitive Complaints Anticipate the Future

‘Just carry‐on living’ was the advice a doctor in a memory clinic gave to one of the interviewees after concluding his complaints did not justify an AD dementia diagnosis. Our study shows that this is easier said than done; for the interviewees ‘carry‐on living’, means living with a double‐edged sword: individuals are not diseased in the medical sense of the word, but their complaints (the ‘illnesses’) are unmistakably there. The experiences of the interviewees align in this regard with those of individuals suffering from ‘Medically Unexplained Symptoms’ (MUS) [27]. Our research shows that living with unexplained cognitive complaints also implies extensive uncertainty about the future, since a defined illness trajectory on which to anticipate is lacking.

The combination of persistent complaints and uncertainty can be understood as an important driver for the ‘scenario‐thinking and ‐acting’ reported in the interviews: individuals suffering from unexplained cognitive complaints keep multiple options open, including a possible future with AD dementia, a scenario which appears to be strikingly dominant for them although none of the interviewees has any medical reason to think that they are ‘at increased risk’. The persistent complaints and uncertainty may also explain the ambivalence in their attitude and actions, reporting they accept whatever the future may bring, but at the same time actively trying to reduce the chance of a future with AD dementia. Here we see what Adams et al. [14] called ‘optimization practices’, in which people—not knowing what will happen—do as much as possible in the present to ensure the *best possible future*. Those practices may become more prevalent when there are strong societal norms about ‘good health behavior’—for example, the increasing tendency to assign responsibility for optimising cognitive health outcomes to the individual and of current societal injunctions to anticipate and prevent the onset of AD dementia—which generate feelings of guilt in case of inaction [28, 29].

### 4.2 The Value of Prognostic Tests for People Living With Unexplained Cognitive Complaints

The interviewees understand ‘biomarker‐based prognostics’ as having the function of ‘predictive diagnostics’, predicting whether one will experience complaints and symptoms *justifying a future AD diagnosis*. The majority of our respondents are positive about this specific idea of biomarker‐based prognostication, which is understandable in view of the uncertainty they experience, having symptoms but not a diagnosis. They also mention *very specific* types of information that would be valuable: what exactly will change mentally or physically, when this will happen, how severe their complaints and symptoms will be, which type of symptoms they will develop, how they will function accordingly and what that would mean for their lives, behaviour and well‐being.

Although research into prognostic biomarkers serves various goals, our study highlights the need to closely monitor whether outcomes measured in such research align with those deemed relevant by people living with unexplained cognitive complaints. Predicting the risk of conversion to MCI and/or AD may address these individuals' need to know what explains their symptoms—but only if such test results would have a high degree of certainty. Moreover, predicting a future diagnosis will hardly help them anticipate how their lives and relationships will be affected, let alone address voiced needs such as timely care planning or decisions whether and when to put in a request for euthanasia. It is questionable whether such tests should be offered if they do not align with the target group's needs. At the very least, our findings emphasise the importance of clear expectation management for individuals and their partners. Or, as one respondent adds in response to our initial findings: ‘because the human body is inherently unpredictable it is of utmost importance to clearly explain to people what the uncertainty of those tests entails.’ Furthermore, based on our findings, individuals considering whether or not to use a prognostic test might be supported in this choice by a conversation on how such tests relate to their attitudes, beliefs and (former) ways of living their life.

For medical professionals and test developers in the domain of AD, it is important to realise that any novel prognostic test offered to people living with medically unexplained cognitive complaints will interfere with their experience of pervasive uncertainty. Individuals suffering from MUS are known to often engage in extensive information‐seeking and testing to understand their symptoms [27, p. 1168], [30, p. 772], [31, p. 113], [32]. Biomarker tests may become part of this (sometimes fruitless) ‘information work’ [27, p. 1169] but may not reduce their uncertainty. Even if people with unexplained cognitive complaints learn they are unlikely to develop AD dementia, they may still face uncertainty about their current condition. As biomarker‐based prognostics are unlikely to eliminate all uncertainty, healthcare professionals should not give the impression they will. Our findings highlight the importance of pairing biomarker prognosis with professional guidance in handling uncertainty. This conclusion was confirmed when we consulted the interviewees about our preliminary conclusions. Only one of them indicated that ‘knowing it is probably not AD dementia’ suffices to remove most uncertainty. Another respondent pointed out that research into and assistance on how to meaningfully cope with this uncertainty might therefore be at least as relevant as research into prognosis and prognostic tools.

Finally, our study raises concerns about the participation of individuals with unexplained cognitive complaints in cohort studies. For those living with MUS, the lack of a diagnosis often leads to diminished expectations about medical possibilities and prompts alternative coping strategies [32]. By participating in the cohort study, however, our interviewees were kept ‘in the loop’ of the biomedical domain. They are invited as ‘healthy subjects’ and informed that their participation is for scientific, not for personal benefit, but in practice, they experience participation as an opportunity for medical and self‐monitoring of complaints. This might hinder their search for other explanations or coping strategies. While two of our respondents noted that participation did not stop them from seeking non‐medical explanations, we suggest considering how to reduce this risk of research participation before inviting this group to regular testing in the context of scientific studies.

### 4.3 Limitations of This Study and Perspectives for Future Research

Selection bias may have been introduced as the interviewees, all participants in a cohort study willing to undergo PET scans for biomarker research, may have a relatively positive attitude towards medical testing. Additionally, it would be interesting to explore whether the strong focus on AD dementia as a future scenario is biased, as this group is annually tested for signs of neurodegeneration.

Biomarker‐based prognostication is not yet available in clinical practice, meaning our respondents had to consider possible futures while uncertain about the cause of their complaints. This, first of all, means we may have been contributing *ourselves* to their ‘information work’ and hope for a medical test to explain their symptoms. Moreover, their specific responses to the specific functions of biomarker prognostics may stem from the difficulty of imagining such an abstract future. However, the hypothetical nature of our questions also allowed participants to freely consider relevant prognostic information.

## 5 Conclusion

In sum, people living with unexplained cognitive complaints clearly value the idea of a test that would *predict* with high certainty whether they will be *diagnosed* with AD dementia in the future. It should be noted, however, that a negative predictive test will not remove the uncertainty about the nature and causes of their symptoms. Regarding *prognostic* tests predicting how their complaints will develop, most but not all individuals suffering from unexplained cognitive complaints expect that such tests could help them and their partners to prepare for the future. However, there is a discrepancy between the (general) outcomes that tend to be measured in prognostic research and the (very specific) outcomes meaningful to our interviewees. It is important to reconsider whether and how the prognostic biomarker tests in development can serve the needs of people living with unexplained cognitive complaints.

## Author Contributions

**Anne‐Fleur van der Meer:** conceptualisation, investigation, writing – original draft, writing – review and editing, methodology, supervision, project administration, formal analysis. **Marjan Knippenberg:** investigation, methodology, writing – review and editing, project administration, data curation, resources, formal analysis. **Denise Visser:** writing – review and editing, resources, data curation, project administration. **Marianne Boenink:** conceptualisation, investigation, writing – review and editing, writing – original draft, project administration, methodology, formal analysis, supervision.

## Ethics Statement

The Institutional Review Board (METC) of the VU Medical Centre, the Netherlands, declared that the interview study on which this paper is based was not subject to the Dutch law on medical research with humans (WMO) and approved the research under file number 2021.0558.

## Consent

All interviewees provided written informed consent before the interview.

## Conflicts of Interest

The authors declare no conflicts of interest.

## Data Availability

The datasets analysed during the current study are not publicly available for privacy protection reasons, but are available from the corresponding author upon reasonable request.

## Endnotes

1 In the literature on individuals living with unexplained cognitive complaints, there is confusion about the appropriate terminology to use when examining the future development of symptoms in these individuals. Because these individuals are not diseased in the biomedical sense of the word, strictly speaking, we should use the term ‘prediction’ (see Box 1). However, because the medical literature dedicated to biomarkers for people living with unexplained cognitive complaints (in that literature these people are referred to as suffering from SCD) mostly uses the term ‘prognosis’ and because this terminology was also used in the larger study our research was part of, we will use the terms ‘prognosis’, prognostics and ‘prognostication’ in this paper.

2 We note that all interviewees live in the Netherlands, where euthanasia is a legal option under strict conditions. Under Dutch law, dementia can be a ground for granting a euthanasia request, but the request must be submitted when the patient is still cognitively competent to do so.
